# Unveiling Non-isothermal Crystallization of CaO–Al_2_O_3_–B_2_O_3_–Na_2_O–Li_2_O–SiO_2_ Glass via *In Situ* X-ray Scattering and Raman Spectroscopy

**DOI:** 10.1021/acs.inorgchem.2c00387

**Published:** 2022-04-25

**Authors:** Shubo Wang, Ekta Rani, Francis Gyakwaa, Harishchandra Singh, Graham King, Qifeng Shu, Wei Cao, Marko Huttula, Timo Fabritius

**Affiliations:** † Nano and Molecular Systems Research Unit, University of Oulu, Oulu FI-90014, Finland; ‡ Process Metallurgy Research Unit, University of Oulu, Oulu FI-90014, Finland; § Canadian Light Source, 44 Innovation Blvd., Saskatoon, Saskatchewan S7N 2V3, Canada

## Abstract

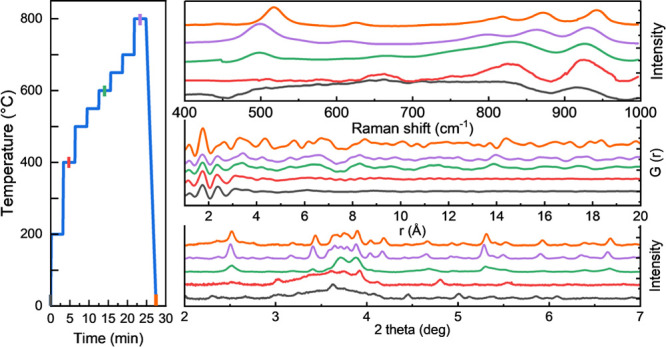

The crystallization in glasses is a paradoxical phenomenon and scarcely investigated. This work explores the non-isothermal crystallization of a multicomponent alumino-borosilicate glass via *in situ* high-energy synchrotron X-ray diffraction, atomic pair distribution function, and Raman spectroscopy. Results depict the crystallization sequence as Ca_3_Al_2_O_6_ and CaSiO_4_ followed by LiAlO_2_ with the final compound formation of Ca_3_B_2_O_6_. These precipitations occur in a narrow temperature range and overlap, resulting in a single exothermic peak in the differential scanning calorimetry thermogram. The concurrent nucleation of Ca_3_Al_2_O_6_ and CaSiO_4_ is intermediated by their corresponding hydrates, which have dominantly short-range order. Moreover, the crystallization of LiAlO_2_ and Ca_3_B_2_O_6_ is strongly linked with the changes of structural units during the incubation stage in non-isothermal heating. These findings clarify the crystallization of multicomponent glass, which have been inferred from *ex situ* reports but never evidenced via *in situ* studies.

## Abstract 2

This work explores the non-isothermal crystallization behaviors of multicomponent alumino-borosilicate glasses via *in situ* high-energy synchrotron X-ray diffraction, atomic pair distribution function, and Raman spectroscopy.

## Introduction

The subject of controlled crystallization within a glass matrix has received intense attention in the glass-crystalline and crystalline materials’ production field due to the feasibility of regulating the glasses’ properties.^1,2^ In the steel industry, glassy materials are employed as mold fluxes to improve the efficiency and smoothness of continuous steel casting and the surface quality of the steel products.^3−5^ Crystallization of such mold fluxes contributes to different heat transfer behaviors between crystalline and glassy mold fluxes.^3^ This reduces the heat transfer between the mold and crystalline layer of mold fluxes, leading to mild cooling of the solidified shell and suppressing uneven solidification in the mold.^6^

The mold flux glasses for casting carbon steels are mainly based on a CaO–SiO_2_–CaF_2_ system^7−10^ wherein CaF_2_ controls the viscosity, crystallization, and melting temperature of the fluxes during continuous casting.^11^ However, volatile and soluble fluorine (F) results in the corrosion of casting facilities and environmental pollution.^4^ The F-free mold flux can be achieved by alterative components involved, such as B_2_O_3_, Li_2_O, and Na_2_O.^12,13^ Additionally, during casting of the new third-generation advanced high-strength steels,^14^ their relatively high Al content leads to a gradual increase in Al_2_O_3_ but a decrease in SiO_2_ in the CaO–SiO_2_-based flux owing to the strong reaction (4[Al] + 3SiO_2_ = 2Al_2_O_3_ + 3[Si]) at the steel melts/fluxes interfaces.^15,16^ These dynamic compositional changes in fluxes would cause reduced lubrication, non-uniform heat transfer, and consequently poor casting efficiency. New mold fluxes based on alumino-borosilicate (CaO–Al_2_O_3_–B_2_O_3_) glasses have been proposed to suppress the oxidation–reduction reaction between SiO_2_ in mold flux and [Al] in steel melts at casting temperatures.^17^

In contrast to the well-documented crystallization characteristics of conventional silicate glasses,^18−20^ crystallization kinetics of alumino-borosilicate glasses is not well understood. Meanwhile, most of the research on the crystallization of glasses mainly focuses on stoichiometry glasses with only a few components,^21^ whereas mold flux glasses contain more than five components to tune the properties and behaviors (viscosity, melting, and crystallization).^8,22,23^ For example, alkali, mainly Li and Na oxides added into the glasses, provides free oxygen in the glass network and decreases the viscosity. The tendency of crystallization of such glassy fluxes is high, and the crystalline products usually have variations in compositions compared with the glass matrix. These emerging factors require profound knowledge about crystallization mechanisms and kinetics for controlling crystallization and achieving a comprehensive application of the mold fluxes in the steel industry.

In this work, the crystallization of a complex alumino-borosilicate glassy mold flux has been probed via combinative high-energy synchrotron X-ray diffraction (HE-SXRD), atomic pair distribution function (PDF), and Raman spectroscopy in the *in situ* modes. Temperature-dependent material variations in the glassy matrix are estimated by differential scanning calorimeter (DSC) thermograms, a commonly used method to reveal the boundary between supercooled liquid and solid (*T*_g_, glass transition temperature) and crystallization and melting characteristic temperatures. The crystallization of phases and the corresponding structural arrangement in short- and medium-range orders are determined by HE-SXRD and PDF, respectively. Information and correlations about the chemical structure are also provided and established by Raman spectrometry. This *in situ* study explores the crystallization mechanism during the non-isothermal annealing process.

## Experimental Section

### Material Synthesis

The investigated glass composition, shown in Table 1, was chosen from a previous work.^24^ The raw materials used to fabricate this glass were analytic-grade CaCO_3_ (>99%), α-Al_2_O_3_ (>99%), H_3_BO_3_ (>99.95%), Na_2_CO_3_ (>99.9%), Li_2_CO_3_ (>99.9%), and SiO_2_ (>99%) supplied by Sinopharm Group Co. Ltd. After removing moisture and thorough mixing, mixtures of raw materials were transferred to a Pt crucible and melted at 1723 K for 2 h. The melts were quenched into the water to obtain the glassy sample. The sample was stored in an ambient environment to follow the application conditions.

**Table 1:** Composition of the Investigated Glass

	CaO	Al_2_O_3_	B_2_O_3_	Na_2_O	Li_2_O	SiO_2_
wt %	37.0	34.0	8.0	8.0	8.0	5.0
atom %	26.4	33.3	11.5	7.7	16.1	5.0

### DSC Measurements

To determine crystallization behaviors, the transformation temperatures were first determined by DSC measurements using a thermal analyzer (STA 449 F3, Netzsch-Gerätebau Gmbh) with Ar gas as the purge gas. Temperature and sensitivity calibration was performed before DSC measurements using high-purity metals with known melting points as reference materials. DSC signals were obtained by heating the glass powder samples to 1400 °C at a heating rate of 20 °C/min. Pt crucibles with a Pt lid were employed to minimize the loss of volatile materials, and high-purity alumina powder was used as the inert reference material.

### *In Situ* HE-SXRD and PDF

**Figure 1:**
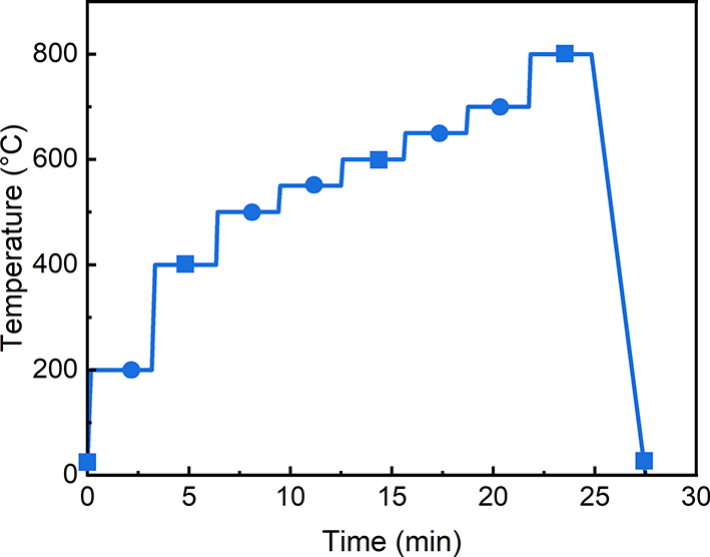
Schematic temperature regime for *in situ* measurements. The circles highlight the temperatures where the HE-SXRD patterns were collected, while the cubes represent the temperatures where both the HE-SXRD and PDF spectra were collected.

The *in situ* HE-SXRD and *in situ* PDF of the powder glass were carried out at the Brockhouse High Energy Wiggler Beamline,^25^ Canadian Light Source, Canada. The refined wavelength of the monochromatic focused beam from standard Ni calibrants was 0.1771 Å. A 2D Perkin Elmer area detector (200 × 200 μm^2^ pixel size, 40 × 40 cm^2^ in area) placed behind the sample allowed XRD and PDF data acquisition in transmission mode. The detector to sample distance was found to be 1230 mm for HE-SXRD and 160 mm for PDF, which allowed a *Q*_max_ of 17 Å^–1^. An exposure time of 2.0 s was used, and the X-ray beam size was approximately 100 μm vertical and 200 μm horizontal. The powder glass sample, at ambient atmosphere, was heated using a flow-cell furnace.^26^ In this case, the powder glass sample was quickly heated from room temperature (RT) to 800 °C with a ramp rate of 20 °C/s in a stepwise manner, as shown in Figure 1. During the heating process, the fast ramp rate was employed to minimize the additional nucleation or crystal growth. At each temperature, the sample was stabilized for 3 min for data acquisition. The data were processed using GSAS-II software.^27^

The percent crystallinity is determined by the ratio of the integrated intensity of the diffraction pattern from the crystalline peaks to the total scattered intensity after subtracting the background scattering^28^
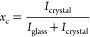
1

where *x*_c_ is the percent crystallinity (wt %), *I*_crystal_ is the integrated intensity of all crystalline peaks, and *I*_glass_ is the integrated intensity of the amorphous glass scattering. The weigh fraction of the crystalline phase is calculated using^29^
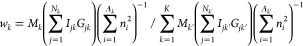
2where *w_k_* is the weight percentage of the *k*th component, *I_jk_* is the integrated intensity of the *j*th diffraction line of the *k*th component, *N_k_* is the number of observed diffraction lines in the defined 2θ range, *n_i_* is the number of electrons belonging to the *i*th atom in the chemical formula unit, *M_k_* is the chemical formula weight, and *G_jk_* is given by *G_jk_* = 2 sin θ_*jk*_ sin 2θ_*jk*_/(1 + cos^2^ 2θ_*jk*_).

### *In Situ* Raman Spectroscopy

The structural analysis by PDF can be qualitative, and a more definitive analysis method can supplement the obtained results. Using the *in situ* Raman measurement, the change in the degree of polymerization based on the alumino-borosilicate glassy mold flux network was further assessed. A confocal micro-Raman spectrometer, with an excitation wavelength of 532 nm, from B&W Tek with a rotatable stage was used for this study. The *in situ* Raman spectra were collected at RT and elevated temperatures (same as the one used for PDF) in the spectral range of 100–2000 cm^–1^. Spectral deconvolution was done using Peakfit software, employing a minimum number of bands to achieve optimum peak fitting. A Gaussian line shape was used to fit the peaks associated with the different structural units; the intensities and widths were unconstrained and independent.

## Results

### DSC

**Figure 2:**
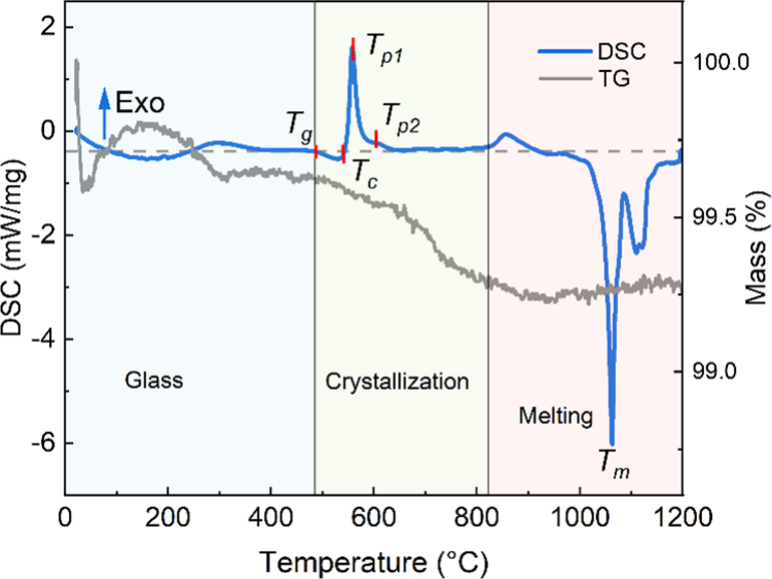
DSC and TG curves of the investigated mold flux glass powder (heating rate: 20 °C/min).

The DSC traces with the thermogravimetric (TG) curve from the as-prepared powder glass, shown in Figure 2, can be divided into three different sections: the glass zone (room temperature to 505 °C), crystallization zone (∼505–820 °C), and melting zone (>820 °C). The powder remains in a glassy state until ∼505 °C. At the low-temperature glass zone, fluctuations of the DSC traces are mainly associated with the relaxation below the glass-transition temperature and dehydration of the as-quenched glass.^30,31^ Afterward, the endothermic glass-transition reaction in the DSC curve is due to a change in the heat capacity attributed to the transformation of glass structures,^2^ and the corresponding glass-transition temperature is located at around *T*_g_ = 505 °C. Followed by, a robust exothermic peak at *T*_p1_ = 558 °C (peak crystallization temperature) is seen, corresponding to the formation of crystalline phases. The onset of the crystallization temperature is estimated as *T*_c_ = 542 °C. It should be noted that there is also a shoulder exothermic peak at *T*_p2_ = 604 °C, indicating minor crystallization events. A weak but long tail follows this crystallization peaks until melting. The small exothermic peak, prior to the melting peaks is most probably connected with a small fraction of less perfect crystals formed after crystallization. These events, seen in multicomponent glasses,^32^ indicate incomplete crystallization before melting. This powder flux exhibits multiple melting peaks with the deepest at ∼1062 °C, showing a multi-stage melting behavior that is frequently observed in glass materials with multiple components.^33,34^ As the investigations in this work were focused on the crystallization process, the temperature range for *in situ* studies was set from RT to 800 °C.

### *In Situ* HE-SXRD

**Figure 3:**
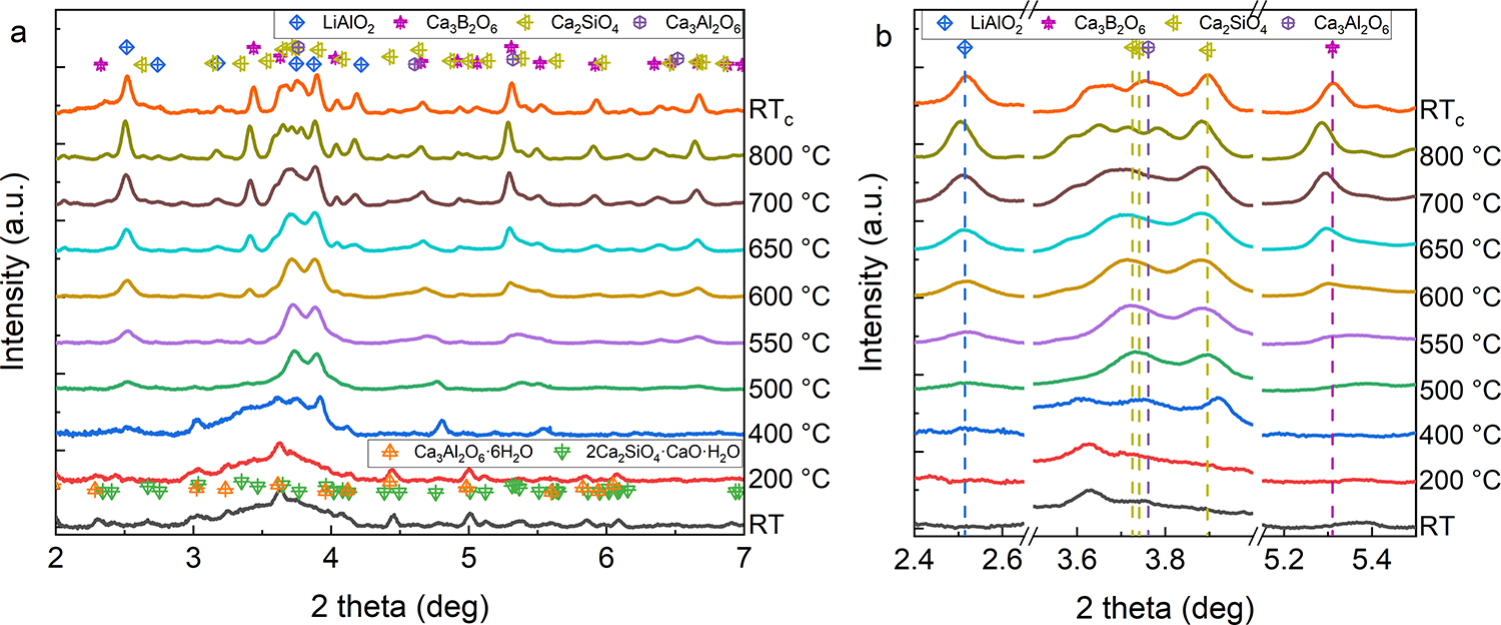
(a) Integrated 1D HE-SXRD profiles acquired during the *in situ* heating experiment at different temperatures; (b) magnified view of the 1D HE-SXRD profiles, in which the dash lines highlight the positions of the most substantial diffraction peak of crystalline phases. The patterns were indexed by referring to the ICDD PDF4+ database.^40^

**Figure 4:**
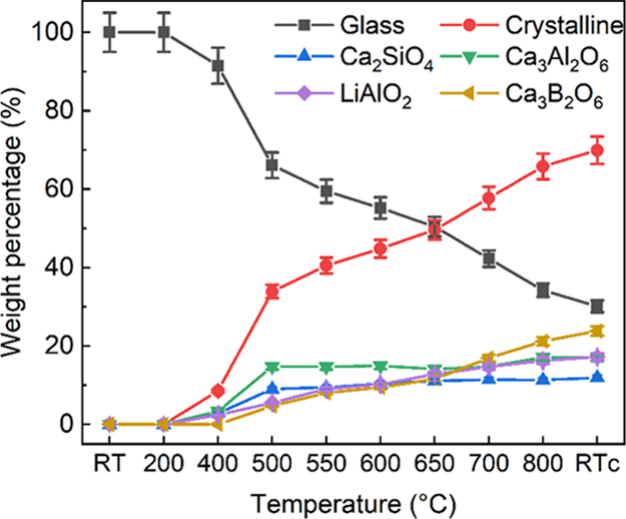
Weight fraction of phases as a function of temperature, estimated by quantitative analysis of HE-SXRD patterns.

**Figure 5:**
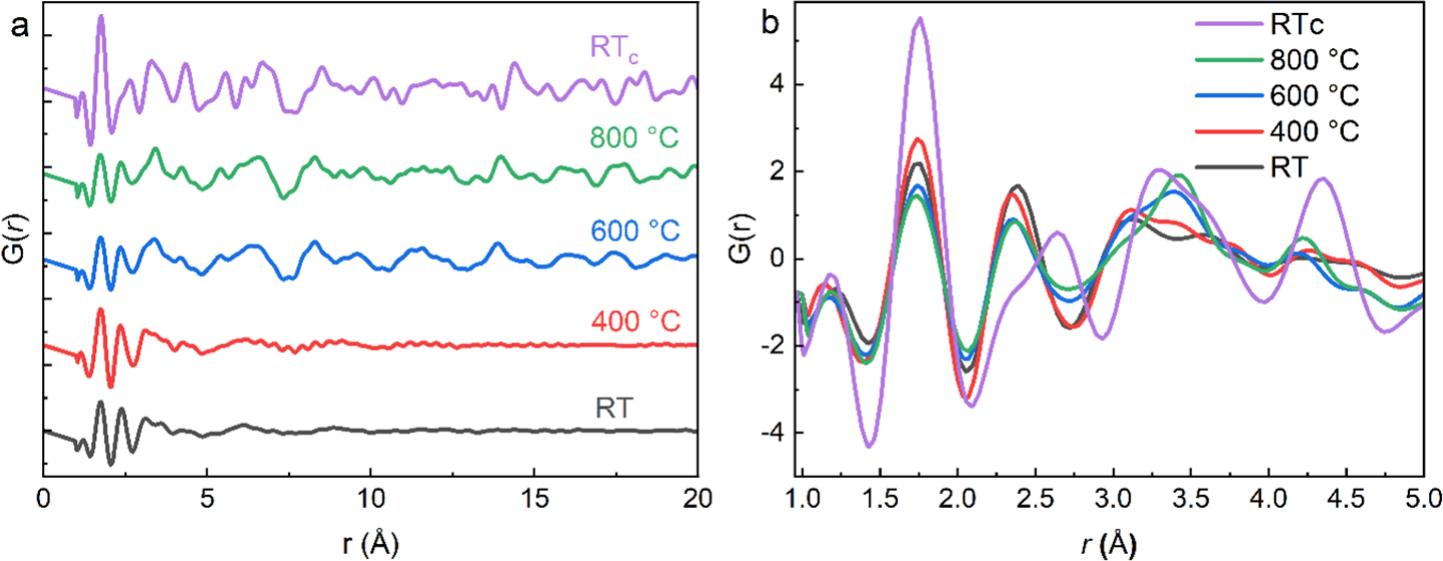
*In situ* PDF data for a sample showing possible distributions of all atom–atom distances at selected temperatures. (a) *r* range from 1 to 20 Å and (b) *r* range from 1 to 5 Å.

Figures 3 and 4 show the 1D integrated HE-SXRD profiles acquired on the sample and determined phase fractions at different temperatures during the *in situ* heating experiment. At RT, the diffraction pattern consists of a broad hump from 2.8 to 4.2° from the glassy matrix. It should be noted that some sharper but still hump-like diffraction peaks also show up, which can be indexed to cubic calcium aluminate hydrate Ca_3_Al_2_O_6_·6H_2_O (ICDD PDF 00-024-0217) and monoclinic calcium silicate hydrate 5CaO–2SiO_2_–H_2_O or 2Ca_2_SiO_4_·CaO·H_2_O (PDF 04-012-5147), which are commonly seen and developed rapidly in cement.^35−37^ In this case, the water sources might be (i) free water standing at the powder flux surfaces and pores due to the long-time storage in the ambient atmosphere, (ii) water absorption in pores, and (iii) Si–OH and Al–OH chemically bounded groups.^38^ It must be pointed out that these hydrate phases are much closer to glass than that to the crystal, as shown in the PDF curve at RT in which short-range (<5 Å) order, less medium-range order, and long-range (>10 Å) disorder are seen (Figure 5). The formation of such disordered species may be due to the clustering of Al/Si and Ca in the glass matrix.^39^

Dehydration of free-standing and absorbed water occurs at relatively low temperatures, while a higher temperature is required to break the bonding of Si–OH and Al–OH.^38^ No significant change has been observed when heating to 200 °C. The hydrates start to dehydrate in the temperature range from 200 to 400 °C as the peaks from crystalline cubic Ca_3_Al_2_O_6_ (PDF 00-008-0006) and monoclinic Ca_2_SiO_4_ (PDF 01-080-8935) are visible at 400 °C. There is also the emergence of a weak peak corresponding to a tetragonal LiAlO_2_ (PDF 01-085-3652). When the temperature reaches 500 °C, signals from hydrate phases disappear entirely, and a clear crystalline LiAlO_2_ shows up. Here, the crystallization onset temperature is different from the one determined by DSC, which could be attributed to the different applied heating rates and the holding time at specific temperatures for data acquisition. At 550 °C, peaks corresponding to trigonal Ca_3_B_2_O_6_ (PDF 00-026-0347) turn up. Afterward, the relative intensity of crystalline peaks increases gradually with the increasing temperature, while the broad hump due to the amorphous glassy matrix tends to disappear simultaneously, implying that the degree of crystallinity increases in Figure 4. Quantification of the fractions of crystalline phases shows that both Ca_2_SiO_4_ and Ca_3_Al_2_O_6_ increase sharply at 400–500 °C, after which the Ca_3_Al_2_O_6_ fraction levels off until 700 °C followed by an increase at 800 °C. While the fraction of Ca_2_SiO_4_ increases gradually until 650 °C and then levels off. A gradual increment of LiAlO_2_ and Ca_3_B_2_O_6_ is observed after 400 and 500 °C, respectively. After cooling down to RT, only right shifts of peaks are detected due to the thermal contraction of the crystalline lattices.

### *In Situ* PDF

The PDF of the powder flux at representative temperatures is plotted in Figure 5. The PDF, *G*(*r*), depicts the distributions of all atom–atom distances in the sample and can provide information on the *in situ* changes in the local structures of an amorphous/crystalline material.^23^ As seen in Figure 5a, short-range order up to <5 Å and long-range disorder >10 Å are clear for the flux up to 400 °C. Weak structural correlations are seen in the medium range (5–10 Å). The PDF data do not allow conclusions associated with the disposition of water or Al(Si)–OH hydroxyl groups as the water content present may be too small to detect using the PDF method.^41^ Nevertheless, the atomic order at the medium-length scale of crystalline hydrate phases in the HE-SXRD pattern is amorphous.

With increasing temperatures, an apparent increment of medium- and long-range order is visible along with increasing temperatures, as shown by the well-defined peaks in *G*(*r*) beyond *r =* 5 Å. The most intense feature in all PDFs in Figure 5b is a sharp peak at 1.74 Å, which is attributed to the Al–O and Si–O bonds that have similar bond distances. Bond valence considerations indicate that the average bond length for Al with coordination numbers of 4, 5, and 6 should be 1.76, 1.84, and 1.91 Å, respectively, suggesting that Al is tetrahedral primarily in the sample at all temperatures. The Si–O bond length for Si in [SiO_4_]^4–^ tetrahedral coordination should be around 1.61 Å.^42^ It is difficult to distinguish the Al–O and Si–O bonds in this study because this requires data collection at cryogenic temperatures, high resolution, and careful peak deconvolution;^42^ however, the PDF data here is collected at room and elevated temperatures. Hence, this significant Al(Si)–O peak at all temperatures is attributed to the higher density of tetrahedrally coordinated Al(Si)–O bonds due to the formation and stabilization of crystalline Ca_3_Al_2_O_6_, LiAlO_2_, and Ca_2_SiO_4_.

The next intense peak at ∼2.37 Å can be attributed primarily to Ca–O bond lengths, although the nearest neighbor B–Al and B–B distances may also contribute to a much smaller extent. The intensity of this peak decreases gradually with the increase in temperature from RT to 800 °C. At RT_c_, after cooling down, the intensity of this peak decreases sharply, and another peak at ∼2.63 Å appears. In Ca_3_Al_2_O_6_, whose formation is evident from the *in situ* HE*-*SXRD, the tetrahedral [AlO_4_] rings are connected by Ca^2+^ ions. As per the literature,^43^ six different Ca sites exist. Three of them, Ca(1–3), form subcell (A), and Ca(4–6) form subcell (B). All calcium atoms in the framework (A) are coordinated to six oxygen atoms with the average Ca–O bond lengths of 2.33, 2.39, and 2.35 Å for Ca(1), Ca(2), and Ca(3), respectively. Calcium atoms forming the framework (B) occupy somewhat irregular environments, and the average Ca–O distances in framework (B) are longer than for framework (A). Ca(4) atoms coordinated by nine oxygen atoms have an average Ca–O distance equal to 2.69 Å, while Ca(5) atoms coordinated by eight have an average Ca–O distance of 2.62 Å, and Ca(6) atoms are coordinated by seven oxygen atoms with an average Ca–O distance of 2.52 Å. A close resemblance of the observed bond distance of 2.63 Å with the literature indicates that the Ca atom is dominantly coordinated by eight O atoms in the studied flux sample after crystallization. The increase in width of these intense peaks indicates a broader distribution of Al–O and Ca–O bond lengths, suggesting that the polyhedra become less regular with the increase in the temperature. This trend can be linked to the increase in polymerization. An AlO_4_/Ca–O_*x*_ with several terminal O atoms can quickly form a nearly ideal polyhedron; however, as it becomes connected to more other polyhedra, the tetrahedron will need to distort to connect to them all.

A weak peak observed at about *r =* 1.2 Å can probably be assignable to the B–O and possible O–H correlations. In boron glasses with high CaO content, B atoms tend to present in [BO_3_]^3–^ trigonal coordination forming boron-oxygen triangles.^44^ The value of *r* is 1.369 Å for B–O bonds in the literature.^45^ Thus, interpreting this peak is very tricky and challenging due to the low contribution of B–O bonds to the PDF, potential overlap with low-*r* termination noise, and with possible O–H bonds.

### Raman Spectroscopy

**Figure 6:**
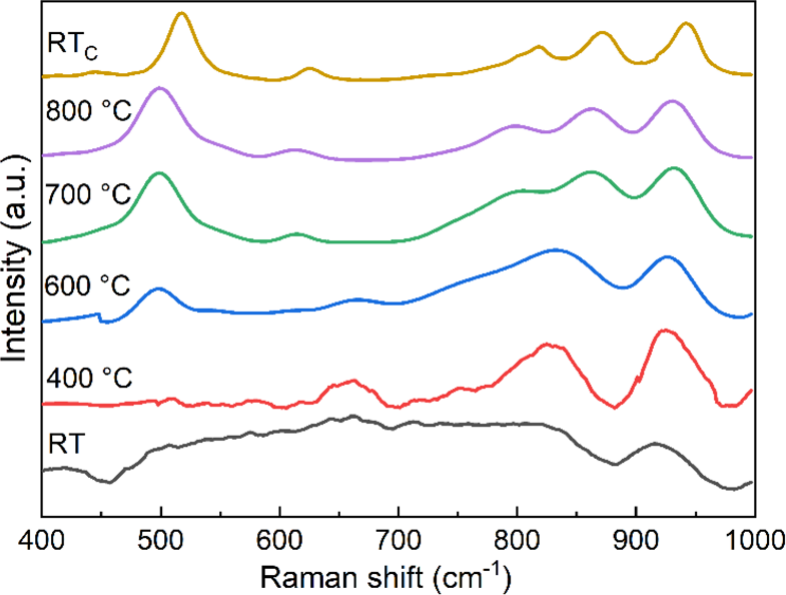
*In situ* high-temperature Raman spectra for the powder flux at different temperatures.

The Raman spectra of the mold flux glass are shown in Figure 6. The Raman spectra consist of dominant vibration bands in three different frequency regions, *i.e.*, a low-frequency region between 450 and 550 cm^–1^, the medium region between 750 and 850 cm^–1^, and the high-frequency region of 850–1000 cm^–1^. At RT, the complex broad bands between 450 and 900 cm^–1^ are developed by ring breathing vibrations characteristic of the initial glassy network. As the crystallization progress proceeds revealed by the DSC thermogram, these complex broad bands turn into more well-defined peaks at elevated temperatures.

**Figure 7:**
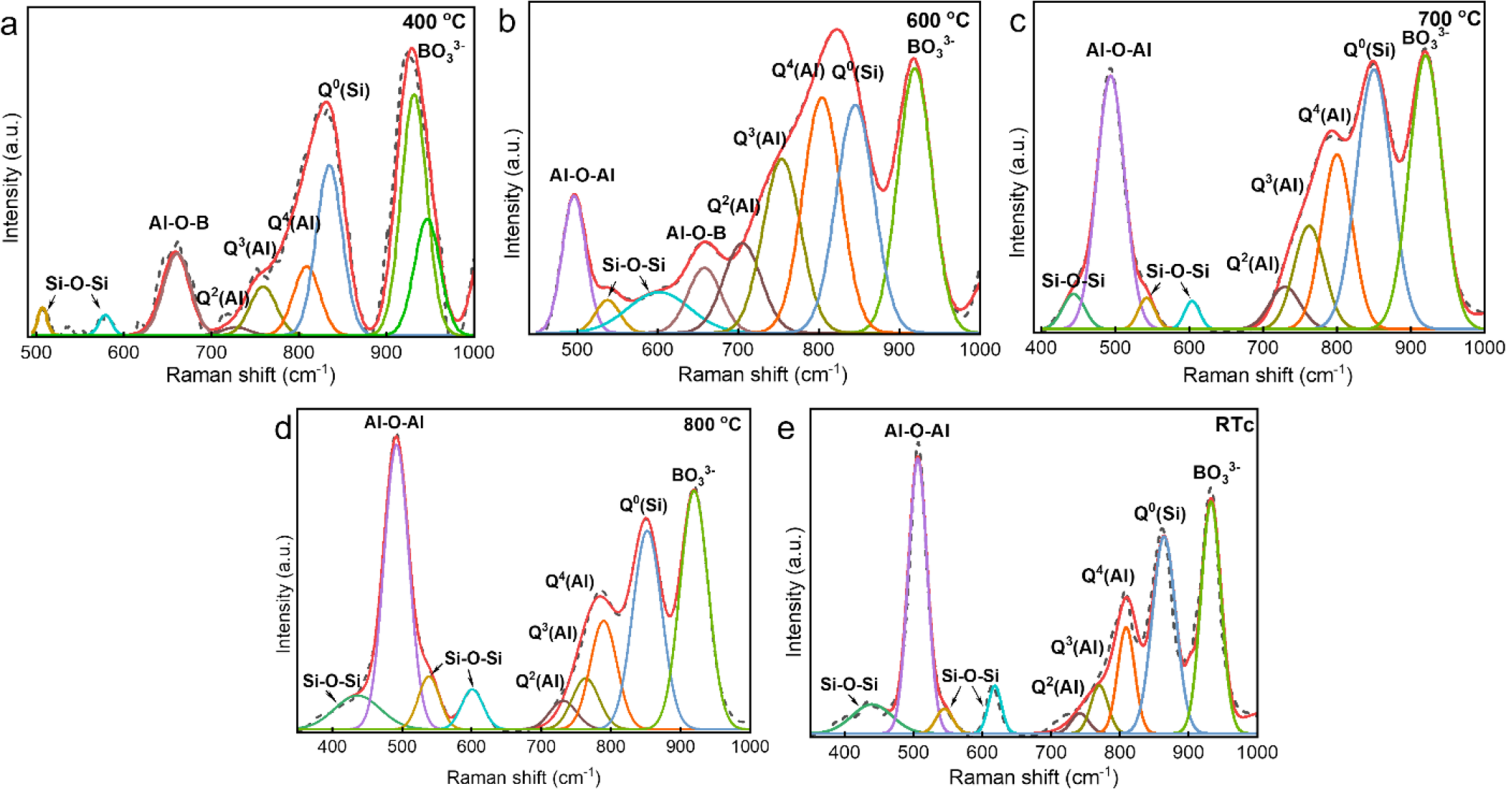
Deconvolution of *in situ* high-temperature Raman spectra for the powder flux at (a) 400 °C, (b) 600 °C, (c) 700 °C, (d) 800 °C, and (e) RT_c_ after cooling down.

After subtracting the background, the spectrum was deconvolved using Gaussian line shapes for contributions from various structural units, as shown in Figure 7. Deconvolution of the Raman spectrum at RT without any crystallization is quite tricky due to the broad hump. Nevertheless, by referencing the 400 °C data (Figure 7a) at which the sample still possesses mainly a short-range ordered glassy structure according to the above HE-SXRD and PDF results, it still can be concluded qualitatively that the initial glassy network structure consists mainly of diffused Si–O–Si linkages associated with silicate-based glass at low frequencies (>600 cm^–1^)^46,47^ and Al–O–B linkages at about 667 cm^–1^^48,49^ and between 650 and 750 cm^–1^, three [AlO_4_] structural units, Q^2^(Al) at 700–735 cm^–1^, Q^3^(Al) at 756–766 cm^–1^, and Q^4^(Al) at 792–802 cm^–1^,^50−52^ and one symmetric stretching Q^0^(Si) of [SiO_4_] tetrahedra at ∼855 cm^–1^ between the region 670–900 cm^-1,^^53^ and the orthoborate [BO_3_]^3–^ group at ∼925 cm^–1^.^54^ As the temperature increases, the Raman bands change in relative intensity. In addition, Raman bands located within the range of 440, 540, and 600 cm^–1^ were observed for the fitted Raman spectra acquired at 600, 700, 800 °C, and RT_c_. These bands could be linked with the Si–O–Si bending vibration of silica in the glass matrix.^46,47^

It can be noticed from Figure 7 that an Al–O–Al band in the frequency region 492–508 cm^–1^ turns up at ∼600 °C and gets sharper at higher temperatures. The increased intensity of the peaks associated with Q^2^(Al), Q^3^(Al), and Q^4^(Al) from Al–O framework vibrations along with the temperature can also be observed. Correspondingly, the intensity of the Raman band corresponding to the Al–O–B bending vibration decreases, and the peak center shifts to a lower frequency (650 cm^–1^) during *in situ* measurements. The change from the glassy flux sample could be associated with the crystallization of aluminates, the Ca_3_Al_2_O_6_ and LiAlO_2_ phase,^52,55,56^ as revealed by *in situ* HE-SXRD and PDF.

The intensity variation of the deconvoluted stretching vibration of isolated [SiO_4_] (Q^0^(Si)) Raman bands (in the range of 800–900 cm^–1^) can be attributed to the presence of the crystalline Ca_2_SiO_4_ phase^57^ as the crystallization process continues with increasing temperatures. Similarly, a slight increase in the relative intensity of the band associated with the symmetric stretching vibration of the BO_3_^3–^ orthoborate unit can be linked to the presence of crystalline Ca_3_B_2_O_6_ (Figure 7). The addition of B_2_O_3_ reduces the viscosity of F-free CaO–Al_2_O_3_–SiO_2_-based mold fluxes^17^ by forming boroxol rings with a [BO_3_]^3–^ triangular structural unit. Table 2 summarizes the peak frequency and normalized integrated peak area of relevant bands of crystalline phases from deconvoluted Raman spectra assigned to network (bonds) structures at different temperatures.

**Table 2:** Relevant Structural Unit of Crystalline Phases and Corresponding Peak Frequency *v* (cm^–1^*)* Range, Normalized Integrated Peak Area (int. area), and Its Percentage (%) Obtained from the Deconvolution of Raman Spectra Acquired from the *In Situ* High-Temperature Measurements

		400 °C		600 °C		700 °C		800 °C		RT_c_	
network structure	*v* range (cm^–1^)	int. area	% area	int. area	% area	int. area	% area	int. area	% area	int. area	% area
Al–O–Al	492–508			792	15	1960	29	1671	31	947	34
Q^2^(Al)	700–735	40	4	563	11	423	6	210	4	133	5
Q^3^(Al)	756–766	110	12	1007	19	845	12	378	7	153	6
Q^4^(Al)	792–802	197	22	898	17	976	14	497	9	230	8
Q^0^(Si)	850–857	259	28	854	16	1409	21	1142	21	502	18
[BO_3_]^3–^	921–930	304	33	1082	21	1178	17	1507	28	804	29

## Discussion

The heat treatment during *in situ* HE-SXRD measurement leaves about 50% Ca, 42% Al, 13% B, 51% Li, and 17% Si (in atom %) behind the glass matrix. This result indicates that the crystallization is not yet completed in the interior of the powder glass. For the crystallization incubation stages, the network structural units change, which do not alter the medium- and long-range disorder of the glass matrix. The crystallization is clearly a heterogeneous metastable process that increases the medium- and long-range order. It is apparent from Raman spectra (Figure 7) that the bands of Al–O–Al for LiAlO_2_, [BO_3_]^3–^ associated with Ca_3_B_2_O_6_, and Q^0^(Si) attributable to Ca_2_SiO_4_ get stronger as the sample moves towards a more crystalline structure with increasing temperatures, also evidenced by the *in situ* HE-SXRD (Figure 3).

Based on the observations obtained in this work, the evolution sequence of crystalline phases in this glass over the temperature can be classified into several stages: (1) the incubation stage from RT to 400 °C, formation of (2) Ca_3_Al_2_O_6_ and Ca_2_SiO_4_ due to dehydration, (3) LiAlO_2_, and finally, (4) Ca_3_B_2_O_6_. In the first stage, changes in Raman spectra indicate a certain degree of local configurational rearrangement of the network structural units (Figure 7) without influencing the long-range disordered glassy matrix (Figure 5). Meanwhile, the crystalline Ca_3_Al_2_O_6_ and Ca_2_SiO_4_ are nucleated from their less ordered hydrate counterpart via
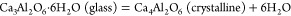
3
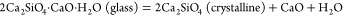
4

The presence of diffraction peaks of the glassy matrix in this temperature range manifests a fairly layered structure of the hydrates, agreeing with the literature.^35−37^ Rapid increase in Ca_3_Al_2_O_6_ and Ca_2_SiO_4_ above 400 °C and the evolution of network structural units shown by Raman spectroscopy (Figure 7) imply that they can also nucleate as
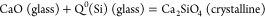
5
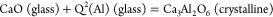
6
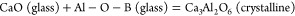
7

The formation of LiAlO_2_ is probably via
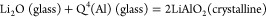
8

The evolution of Ca_3_B_2_O_6_ in this glass is due to the reaction
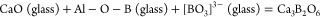
9

An equilibrium among Q^2^, Q^3^, and Q^4^ structural units was found by Mysen et al.^58^ during Raman investigation of binary alkali silicate liquid:
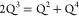
10

This reaction shifts to the right with a temperature increment. It is postulated that in aluminate glass and melts, a similar equilibrium among Q^2^(Al), Q^3^(Al), and Q^4^(Al) persists:
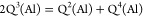
11

The equilibrium shift toward the right side of the reaction and the abundance of Q^4^(Al) could be evidenced by the increased Al–O–Al peak height at high temperatures and the increased area ratio of Q^4^(Al) to Q^3^(Al) from 600 to 800 °C in high-temperature Raman spectra.

In general, the crystallization process is thermally activated and controlled mainly by diffusion from bulk glass–ceramic to the glass–crystal interface. The introduction of alkali oxides, Li_2_O and Na_2_O, is meant to decrease the viscosity and provide more simple structural units, such as 2D [BO_3_]^3–^, Q^0^[SiO_4_], by reducing the degree of polymerization.^48,59^ Such a modification facilitates the diffusion of ions; therefore, this investigated mold flux has a high potential to crystallize. The observed only one strong exothermic peak in DSC is attributed to the overlap of peaks from crystallization of different phases in a quite narrow temperature range, as illustrated by the *in situ* HE-SXRD and PDF analysis.

## Conclusions

The results of this study shed some light on the understanding of the crystallization phenomena in multi-component alumino-borosilicate glass, which make up of steel industrial wastes. The non-isothermal crystallization of the glass is investigated by *in situ* synchrotron X-ray diffraction, PDF, and Raman spectroscopy. The following conclusions can be drawn:

- Crystallization of this glassy flux begins with the formation of Ca_3_Al_2_O_6_ and CaSiO_4_ followed by the precipitation of LiAlO_2_ and Ca_3_B_2_O_6_.
- The nucleation mechanism of Ca_3_Al_2_O_6_ and CaSiO_4_ in the complex glass is triggered by the dehydration of corresponding hydrate phases, which has short-range order but long-range disorder, a fairly layered structure, and limited content in the glass matrix. This finding is further supplemented by the Raman results. Crystallization of subsequent LiAlO_2_ and Ca_3_B_2_O_6_ is strongly related to the changes of structural units during non-isothermal heating.
- The nucleation of this complex multicomponent alumino-borosilicate glass is a heterogeneous metastable process and occurs in a quite narrow temperature range through the precipitation of multiple crystalline phases.
